# Social impact of happiness on transformational leadership in students from Colombian universities

**DOI:** 10.3389/fpsyg.2025.1571572

**Published:** 2025-08-20

**Authors:** Rodrigo Zárate-Torres, C. Fabiola Rey-Sarmiento, Julio C. Acosta-Prado

**Affiliations:** ^1^Department of Research, Colegio de Estudios Superiores de Administración, Bogotá, Colombia; ^2^Department of Engineering, Pontifical Catholic University of Peru, Lima, Peru

**Keywords:** happiness, Subjective Happiness Scale, LPI, confirmatory factor analysis, structural equation model, SEM

## Abstract

In the literature, it has been described that affective commitment and transformational leadership are directly related to occupational happiness. In this sense, the present study begins with the premise that subjective happiness may enhance the effectiveness of transformational leadership. For this purpose, the Leadership Practices Inventory (LPI) and the Subjective Happiness Scale (SHS) were applied to a non-probabilistic sample of 215 business administration students in Colombian universities. Bivariate correlation analysis, confirmatory factor analysis, and structural equation modeling were used to examine the relationship between the dimensions of happiness and the five leadership practices. The findings indicate that three happiness items correlate positively with transformational leadership practices, suggesting that happier individuals exhibit more effective leadership behaviors. The proposed theoretical model showed a good fit with the empirical data. In conclusion, promoting happiness in leaders not only improves their performance but also positively impacts organizational productivity.

## 1 Introduction

Recent studies highlight that perceiving work as meaningful and aligned with personal goals is a key predictor of job happiness. This includes feeling appreciated by colleagues and enjoying daily tasks (Charles-Leija et al., 2023).

Happiness at work improves individual wellbeing and positively impacts organizational productivity, creativity, and innovation. It also reduces turnover intentions and fosters healthy work environments (Cassar and Meier, 2018; DiMaria et al., 2020; Charles-Leija et al., 2023). For the present paper, we will use the definition of Lyubomirsky and Lepper (1999), who developed the Subjective Happiness Scale (SHS) and defined happiness as an overall positive self-evaluation of one's own life. It is a broad construct that integrates emotional, cognitive, and social dimensions. Understanding and promoting it is essential for individuals and organizations (Fisher, 2010). However, in organizations, the leader is in direct contact with the employee, and therefore, the responsibility falls primarily on them and their management style. Leader behavior is essential to employee wellbeing and significantly influences their lives (Harris and Kacmar, 2006). In addition, leaders can promote and generate employee happiness by developing motivation, awareness, and dedication in their followers by engaging in transparent, two-way communication and creating a positive environment (Pangarso et al., 2019).

Scholars have conventionally examined leadership from two angles: one emphasizes positional leadership in the hierarchy of an organization. At the same time, the other sees leadership as a social influence process that emerges spontaneously in a social system (Helland and Winston, 2005). Four approaches to leadership theories have been identified due to these perspectives: transformational, behaviorist, contingency, and characteristics (Helland and Winston, 2005). All of these techniques have examined the traits and actions of leaders, how they utilize influence and power, and how they adapt their conduct in response to unavoidable circumstances, according to Helland and Winston (2005). As per Van Maurik (2001), these four approaches have no mutual exclusion or time-limited determination.

The Leadership Practices Inventory (LPI) is grounded in the theoretical framework of Kouzes and Posner's transformational leadership approach (Kouzes and Posner, 2019; Bass and Riggio, 2006). The approach mentioned above was derived from an extensive review of numerous case studies documenting individuals' personal-best leadership experiences, whereby they achieved remarkable feats (Northouse, 2016; Posner and Kouzes, 1988; Kouzes and Posner, 1990). The theoretical framework posits that there exist five distinct leadership practices or behaviors that are considered exemplary: Modeling the Way, Inspiring a Shared Vision, Challenging the Process, Enabling Others to Act, and Encouraging the Heart (Kouzes and Posner, 1990).

On the other hand, human beings share a common goal of achieving wellbeing and happiness (Lewis et al., 2002). Recent decades have witnessed an explosion in research on happiness, or subjective wellbeing (Lyubomirsky and Tucker, 1998; Urzúa et al., 2009), and it has extended from management areas to mental health (Steel et al., 2008). Happy people succeed across multiple domains of life, including marriage, friendship, income, job performance, and health (Lyubomirsky et al., 2005a). Those who perceive themselves as happy respond more adaptively to everyday experiences, in decision-making, in the perception and interpretation of social situations, and recovery from adverse events such as failure (Lyubomirsky and Tucker, 1998; Lyubomirsky et al., 2005b,a).

This study aims to show the influence of happiness on leaders' effectiveness (model the way, inspire a shared vision, challenge the process, enable others to act, and encourage the heart) and test the premise that “the greater the happiness, the greater the productivity”. To do so, the research question is stated as follows: What is the direct effect of happiness on a leader's effectiveness in improving the productivity of followers? The study employed conceptual frameworks to examine the relationship between happiness scores and leadership-related behaviors. To accomplish this, we selected a total of 215 participants who were currently pursuing graduate and undergraduate degrees in business administration from different universities in Colombia. Apart from continuing this research orientation, the contribution of this paper is visible in terms of principles of open science, as it incorporates reproducible research standards that facilitate the audit of findings and maximize the chances of reproducibility in further studies (Hardwicke et al., 2022).

The study is divided into seven sections. Section 1 presents the context of the study problem, as well as the focus of the objective and the research question. Section 2 defines and integrates the two conceptual frameworks on which the study is based. Section 3 describes the development of hypotheses, stating the hypotheses of the study. Section 4 describes the methodology used, detailing the data collection process. Section 5 presents the results in line with the study's objective. Section 6 discusses the findings, as well as their theoretical and practical implications. Finally, Section 7 presents the conclusions.

## 2 Literature review

### 2.1 Happiness

Throughout history, happiness has been understood from multiple perspectives, ranging from philosophical to organizational and encompassing psychological approaches. In classical Greece, Plato associated happiness with knowledge and virtue as paths to the fullness of the soul (Platón comp. Lan et al., 2016). Centuries later, Russell (1936), in his work 'The Conquest of Happiness,' defined it as an emotional experience associated with affection, enthusiasm, and meaningful relationships. With the advance of psychology in the 20th century, happiness began to be approached from a scientific perspective. Russell (1936) established the modern philosophical basis, while authors such as Lyubomirsky and Tucker (1998) extended the analysis to the study of individual differences in the perception of happiness, proposing that happy people tend to interpret everyday events more positively. Subsequently, Lyubomirsky and Lepper (1999) developed the Subjective Happiness Scale (SHS), which defines happiness as an overall positive self-evaluation of one's life. This scale is based on four items that capture general and comparative perceptions of happiness, as well as positive and negative emotional dispositions. This variable definition will be used for this paper because it includes its operational definition and validated assessment mechanism in Spanish.

Alarcón (2006) contributed to the development of the construct with the proposal of a factorial scale to measure happiness, understood as a subjective state of satisfaction with life and with the material and symbolic goods achieved. Later, Alarcón (2015) reaffirmed this perspective by conceptualizing happiness as a personal experience arising from the achievement of desired goods. From a global and applied perspective, international reports on happiness (Helliwell et al., 2017, 2018, 2020; Rowan, 2023) have proposed that happiness should be considered a key indicator of sustainable development. These reports integrate variables such as social support, freedom of choice, generosity, perceived corruption, and health as dimensions that directly impact the subjective happiness levels of countries.

In the organizational context, Fisher (2010) defines happiness at work as a broad construct encompassing emotional, cognitive, and social dimensions. According to this author, promoting happiness in the workplace enhances individual wellbeing, improves productivity, creativity, and job tenure. Along the same lines, Salas-Vallina et al. (2017, 2020) have developed a line of research focused on happiness at work (HAW), identifying factors such as inspirational leadership, organizational commitment, and social support as key drivers of work wellbeing. These authors also emphasize that happiness at work fosters prosocial behavior, enhances the organizational climate, and improves both individual and collective performance. Finally, the work of Tkach and Lyubomirsky (2006) explores the strategies people employ to improve their wellbeing, reaffirming that happiness is not a static state but a process that can be actively developed through deliberate decisions, habits, and perceptions. This evolution enables understanding happiness as a multidimensional construct that encompasses emotional, cognitive, and social aspects, which are essential for both individual and collective development.

Some scales are theoretically associated with evaluating wellbeing, including happiness. Among others, there are the Satisfaction with Life Scale (Pavot and Diener, 2009), the Short Depression-Happiness Scale (SDHS) (Joseph et al., 2004), the Oxford Happiness Inventory (Lewis et al., 2002), the Steen Happiness Index (SHI) (Seligman et al., 2005), and the factorial scale of happiness (Alarcón, 2006).

The “Subjective Happiness Scale (SHS)” developed by Sonja Lyubomirsky and Heidi Lepper (Lyubomirsky and Lepper, 1999) is used in this study. It is a measure of happiness of 4 items: (1) In general, I consider myself... (2) Compared to most people around me, I consider myself... (3) Some people are usually very happy. They enjoy life despite what happens, coping with most things. To what extent do you consider yourself such a person? 4) Some people are usually very unhappy. Although they are not depressed, they do not seem as happy as they would like. To what extent do you consider yourself such a person (Lyubomirsky and Lepper, 1999)?

This scale has been validated in different languages: Greek (Karakasidou et al., 2016), Mandarin (Chien et al., 2020), Arabic (Alquwez et al., 2021), French (Zager Kocjan et al., 2022), Portuguese (Ortiz et al., 2021; Zanon et al., 2022), Asian countries (Powell et al., 2020), and Spanish, version used in the present study (Extremera and Fernández-Berrocal, 2014; Quezada et al., 2016; Sousa et al., 2017; Feliu-Soler et al., 2021).

### 2.2 Leadership practices inventory—LPI

The conceptual development of leadership has evolved significantly over time, adapting to the organizational, social, and human challenges of each era. One of the most influential milestones in the study of leadership is the distinction proposed by Burns (1978), who introduced the concepts of transactional and transformational leadership. Transactional leadership is characterized by an exchange system based on rewards and punishments, which motivates compliance. In contrast, transformational leadership represents a process in which leaders and followers push each other to achieve higher levels of morale and motivation. This vision marked a significant departure from earlier models focused solely on formal authority. According to more recent research, there is a correlation between transformational leadership and knowledge-sharing behavior from the perspective of collaborators but not from that of supervisors (Durán and Castañeda, 2015). Kouzes and Posner initiated their intensive research project in 1983 to identify the leadership competencies essential for achieving extraordinary results in organizations, grounded in the theory of transformational leadership. Their findings were first identified and presented in their internationally best-selling book, The Leadership Challenge, which was first published in 1987 Kouzes and Posner (1987). At the beginning of the 21st century, authors such as Helland and Winston (2005) described the analysis of leadership by four complementary streams: the behavioral approach (focused on what leaders do), the contingency approach (which values the adaptation of style to context), the trait approach (which examines personal qualities) and the transformational approach. This taxonomy recognizes that no approach is mutually exclusive, as Van Maurik (2001) also argues that these perspectives coexist and intertwine in practice. During this stage, authors such as Bass and Riggio (2006) empirically strengthened the transformational leadership model, highlighting its positive influence on variables including organizational commitment, productivity, wellbeing, and role clarity. Studies over the past five years relate leadership practices in school leaders to the success of school organizations, creating a shared purpose based on opinions, values, and the school's mission (Emmanuel and Valley, 2022) and exploring common attributes of inclusive young leaders, applying the LPI framework and extending it to the five attributes of inclusive young leaders (Ackaradejruangsri et al., 2023).

The LPI evaluates five leadership behaviors that are considered exemplary behaviors of leaders (Kouzes and Posner, 2019; Zárate Torres and Matviuk, 2012). The instrument has been validated in Spanish in different contexts (Robles Francia et al., 2013; Francia et al., 2012; Díaz et al., 2020; Zárate-Torres and Correa, 2023).

These practices consist of: (1) Challenging the process: This approach involves requesting clarification, seeking novel ideas, identifying opportunities, taking calculated risks, and learning from past mistakes (Kouzes and Posner, 2019). (2) Inspiring a shared vision: This strategy emphasizes how the leader communicates or clarifies the vision to his subordinates. When a leader enlists the help of his followers, he may share his vision and his love for it with them (Kouzes and Posner, 2019). (3) Enabling others to act: This approach involves empowering subordinates, encouraging cooperation, and delegating (Kouzes and Posner, 2019). (4) Modeling the way: This approach describes how a leader sets an example by being clear about their principles, being aware of themselves, and being consistent in their actions, words, and style of living out their beliefs (Kouzes and Posner, 2019). (5) Encouraging the heart: This final technique involves the leader's acknowledgment of individual and group accomplishments, both in public and private (Kouzes and Posner, 2019).

### 2.3 The influence of happiness on leadership

Since the late 1990s, the theory of positive psychology has been developed, suggesting that happy people tend to perform better in various areas of life, including work. In this sense, Lyubomirsky and Tucker (1998) observed that subjective happiness influences how social situations are interpreted, decisions are made, and adverse events are faced; in later studies, Lyubomirsky et al. (2005a) expanded this view by pointing out that happy people achieve higher levels of personal success in areas such as marriage, health and, in a complementary way, better work performance. From this approach, it has been argued that leadership is not only a technical practice but also an emotional and human expression directly affected by the emotional state of the leader.

In studies of happiness in the organizational environment, as previously described, Fisher (2010) proposed that happiness at work has a direct impact on creativity, innovation, productivity, and healthy environments. This perspective reinforced the notion that the leader's wellbeing was a determining factor in their style and effectiveness. In line with the above, Blomme (2012); Blomme et al. (2015) highlighted that leaders should provide feedback, autonomy, and social support to foster work happiness, which makes leadership a generating agent of positive emotions within teams.

More recent literature recognizes the role of transformational leadership as a catalyst for emotional wellbeing. Salas-Vallina et al. (2017) demonstrated that leadership focused on inspiring, enabling, and encouraging influences the development of positive attitudes, including job satisfaction and affective engagement.

Likewise, research such as Abdullah et al. (2017) found that affective commitment and transformational leadership are directly related to work happiness. This relationship is enhanced when collaborators possess positive personal characteristics Salas-Vallina et al. (2017, 2020)

In the context of changing societies, more integrative approaches have been developed. Vallina and Guerrero (2018) proposed that servant leadership serves as a facilitator of wellbeing and happiness, highlighting that empathetic, ethical, and people-centered leaders strengthen interpersonal bonds and the organizational climate. In this sense, studies by Srivastava et al. (2022) further explored how spiritual leadership promotes happiness at work from a self-determination theory perspective, thereby fostering prosocial behaviors and team cohesion.

By applying the Subjective Happiness Scale (SHS) and the Leadership Practices Inventory (LPI), we propose an inverse relationship to that analyzed in previous studies. Therefore, this article examines the relationship between happiness and organizational leadership effectiveness. To do so, we establish a correlational model with the Leadership Practices Inventory based on the four items that explore the positive and negative dimensions of happiness (self-perception, perception compared to others, ability to enjoy life, and perceived unhappiness). This allows us to analyze its influence on work dynamics, labor productivity, and the formalization of these mechanisms that contribute to the optimal functioning of people and organizations and the effective management of their wellbeing and development (Lorente and Vera, 2010; Rivera et al., 2018; Olvera-Calderón et al., 2017; Salanova and Llorens, 2009).

Unlike most previous works focused on Anglo-Saxon or European environments, this research is developed in Colombia, offering a situated view that reflects the cultural, social, and economic complexity of Latin America, which represents a contribution to reducing the gap in the literature on leadership and happiness, especially in contexts of emerging economies and countries of the Global South. In a scenario characterized by accelerated organizational transformations, structural inequalities, and development challenges, the findings provide insight into how happiness influences transformational leadership practices, offering relevant and contextualized empirical evidence that has been largely underdocumented in the region. On the other hand, this study defines and operationalizes the concept of happiness within Spanish-speaking organizations by applying the Subjective Happiness Scale (Lyubomirsky and Lepper, 1999), which has been previously validated in Spanish, thereby seeking to account for the cultural relevance of the measurement and its practical applicability. This approach enables the evaluation of leaders' self-perception of happiness, facilitating its integration into organizational development and labor welfare programs.

In contexts such as Latin America, where studies on happiness at work are still incipient, this proposal opens the way for new research. It offers a valuable tool for human talent managers in organizations. Complementary to the above, this study proposes a clear causal model that allows for understanding the link between the leader's happiness and their performance in transformational leadership practices through statistical analysis using structural equation models, which identify significant relationships between emotional and behavioral variables. This model not only empirically validates the proposed hypothesis but also lays the groundwork for future research to investigate mediating variables, such as emotional intelligence, organizational culture, or work commitment, thereby enriching the theoretical framework on effective leadership in changing contexts.

Furthermore, this study seeks to influence empirical research results that favor the emergence of evidence and serve to improve the perception of happiness in organizations and its influence on leadership for the construction of healthy organizations and work models (Salas-Vallina et al., 2017; Ravina Ripoll et al., 2019; Setiawan et al., 2020; Núñez-Barriopedro et al., 2020; Mendoza-Ocasal et al., 2021; Srivastava et al., 2022; Ravina Ripoll et al., 2022; Ravina-Ripoll et al., 2022).

## 3 Hypothesis development

### 3.1 Self-perceived happiness and leadership practices inventory (LPI)

Several studies have demonstrated a positive correlation between perceived happiness and the implementation of effective leadership practices. The literature indicates that happiness at work and leadership influence each other, creating a virtuous cycle that benefits both individual wellbeing (Lyubomirsky et al., 2005a,b) and organizational performance (Rivera et al., 2018; Ravina Ripoll et al., 2022; Ravina-Ripoll et al., 2022). For example, recent research has identified that transformational leadership is associated with higher levels of happiness at work, and that happiness management can contribute to the development of more effective, inspiring, and ethical leaders (López Pérez et al., 2023). From the perspective of Conservation of Resources (COR) theory, the subjective perception of happiness and wellbeing functions as a personal resource that enhances self-efficacy and self-management, both central elements for effective leadership (Junça-Silva and Camaz, 2023). When individuals experience high levels of subjective wellbeing, they tend to demonstrate greater self-leadership capacity, which translates into better leadership practices. Thus, self-perceptions of happiness can facilitate self-organization, self-motivation, and personal monitoring, key competencies assessed by the LPI. Empirically, happiness in the workplace has been shown to enhance productivity, creativity, and commitment–attributes often found in effective leaders (Rienzi, 2025). Furthermore, quantitative studies have found direct correlations between transformational leadership and employee happiness, concluding that happy employees produce better results and greater productivity, which reinforces the hypothesis that the perception of happiness positively impacts leadership practices (Fisher, 2010; Salas-Vallina et al., 2017; Canal et al., 2023). In summary, self-perceived happiness not only increases individual wellbeing but also acts as a facilitator of effective leadership practices, as measured by the LPI. This relationship is explained by both theoretical models and empirical evidence, which supports the proposed hypothesis. Therefore, the following hypothesis is formulated:

*H*_1_*: Self-perceived happiness (HAP1) positively influences the Leadership Practices Inventory (LPI)*.

### 3.2 Happiness perception compared to others and leadership practices inventory (LPI)

The literature suggests that happiness, understood as a subjective perception of wellbeing, has a direct influence on performance and leadership practices. For example, recent research has shown that leaders who report higher levels of happiness tend to adopt more transformational, participatory, and authentic leadership styles, which translates into greater effectiveness in team management and achieving organizational goals (López Pérez et al., 2023; Martínez-Conesa et al., 2024). Furthermore, a leader's happiness is contagious and can influence the team's emotional climate, increasing employee satisfaction and performance (Visser et al., 2013; Institute of Leadership & Management, 2011). On the other hand, comparative studies have found that those in leadership positions tend to report higher levels of happiness and life satisfaction compared to those who do not lead, suggesting a bidirectional relationship between the two variables: happiness favors effective leadership, and, in turn, the exercise of leadership can increase the perception of happiness (López Pérez et al., 2023). Likewise, the study by Martínez-Conesa et al. (2024) supports that positive emotions, especially happiness, influence leadership and decision-making, which is reflected in better leadership practices assessed by the LPI. Finally, evidence shows that the perception of happiness, especially when compared to that of others (social comparison), can strengthen a leader's self-perception of competence and effectiveness, promoting behaviors aligned with leadership best practices, such as inspiration, modeling values, and fostering innovation (Institute of Leadership & Management, 2011; López Pérez et al., 2023). This aligns with the theory of emotional contagion and positive psychology, which postulates that positive emotional states in leaders are transmitted to teams and enhance organizational performance (Visser et al., 2013). Therefore, the following hypothesis is formulated:

*H*_2_*: Happiness perception compared to others (HAP2) positively influences the leadership practices inventory (LPI)*.

### 3.3 The ability to enjoy life and leadership practices inventory (LPI)

Recent literature highlights that leaders who experience high levels of wellbeing and personal enjoyment tend to generate more positive, motivating, and productive work environments (López Pérez et al., 2023). Leaders' positive attitudes are “contagious” and directly impact team climate, job satisfaction, and employee creativity (Sánchez et al., 2021). Thus, a leader who enjoys life conveys optimism and resilience, which is essential for practicing transformational, inspirational, and ethical leadership practices–core dimensions of the LPI (García, 2016). The ability to enjoy life enhances skills such as creativity, resilience, and emotional intelligence, essential competencies for effective leadership (García, 2016). The state of “flow”, described by Csikszentmihalyi (1990), occurs when a person is fully absorbed and enjoying an activity; this state fosters full engagement and performance excellence, qualities reflected in leadership best practices (García, 2016). The literature suggests a causal relationship between positive leadership and employee happiness, particularly in leadership models that prioritize human development and employee wellbeing (López Pérez et al., 2023). Leaders who enjoy their life and work are more likely to practice recognition, empathy, and inspiration, aspects assessed by the LPI (Sánchez et al., 2021; López Pérez et al., 2023). Effective leadership seeks alignment between personal values, purpose, and action. Leaders who find meaning and enjoyment in their personal and professional lives tend to radiate enthusiasm and commitment, which strengthens their ability to influence their teams and achieve sustainable results positively (Salas-Vallina et al., 2017; Sánchez et al., 2021; Ravina Ripoll et al., 2022). Therefore, the following hypothesis is formulated:

*H*_3_*: The ability to enjoy life (HAP3) positively influences the leadership practices inventory (LPI)*.

### 3.4 Perceived unhappiness and leadership practices inventory (LPI)

Several studies have shown that happiness at work is closely linked to the leadership exercised in organizations. Positive and transformational leadership is associated with higher levels of employee wellbeing and happiness, while harmful or toxic leadership styles generate dissatisfaction, demotivation, and lower performance (López Pérez et al., 2023). Therefore, it can be inferred that leaders' perceived unhappiness can negatively affect how they exercise and perceive their leadership practices. The literature indicates that leaders who experience negative emotions or unhappiness tend to exhibit less effective behaviors, such as micromanagement, aggressive criticism, a lack of support, and poor communication, which not only affect the work environment but also hinder their ability to implement effective leadership practices (Kılıç and Günsel, 2019; Wolor et al., 2022). These behaviors can translate into lower scores on the various components of the LPI, as unhappiness can diminish motivation, creativity, and the willingness to inspire and develop others (Kılıç and Günsel, 2019; Wolor et al., 2022). Leaders' psychological wellbeing is a fundamental resource for exercising constructive leadership. When unhappiness is perceived, it leads to a resource drain, resulting in less vigor, greater burnout, and lower affective commitment, negatively affecting both work outcomes and leadership practices assessed by the LPI (Hancock et al., 2021; Wolor et al., 2022). Thus, perceived unhappiness can limit a leader's ability to model the path, inspire a vision, and encourage their team, core dimensions of the LPI. In summary, theoretical and empirical evidence shows that unhappiness affects leaders' motivation, commitment, and ability to perform effective behaviors, which is reflected in low scores on the LPI (Kılıç and Günsel, 2019; Wolor et al., 2022; Hancock et al., 2021; López Pérez et al., 2023).

*H*_4_*: Perceived unhappiness (HAP4) negatively influences leadership practices inventory (LPI)*.

## 4 Materials and methods

Our approach is comparable to earlier research that examined the relationship between leadership qualities and happiness (Vallina and Guerrero, 2018; Srivastava et al., 2022); however, the latter employed a different framework to concentrate on distinct objectives.

Ours is a nonprobabilistic study with convenience sampling. This study uses a cross-sectional research design, which is an observational study that analyzes data from a population or a representative sample at a single point in time or over a specific, limited period. This approach is akin to taking a “snapshot” of a situation at a given instant, allowing for the simultaneous observation of exposure and outcome. Such a design is particularly valuable for hypothesis generation, describing health and disease patterns within a population, and for planning health services (Levin, 2006).

The study's total sample consisted of 241 undergraduate and graduate students of business administration programs from various universities in Colombia. Once the data were curated, 215 students were left, and the data were used to perform the statistical analyses. The 26 students excluded from the study did not complete the instruments, leaving many questions unanswered.

Of them, 47.91 % were women, and 52.09 % were men. 78.60 % of the participants were graduate students, and 21.40 % were undergraduates in their final semester of study. Of the participants, 35.81 % were between the ages of 20 and 25, 41.39 % were between the ages of 26 and 35, 16.74 % were between the ages of 36 and 45, and 5.58 % were older than 46. Data were gathered using legitimate, already-translated questionnaires in Spanish. The lead researcher was always present in the classroom to answer student inquiries and avoid confounding factors that would have complicated questionnaire administration or resulted in problems with uncontrolled statistical variance (Rodríguez-Ardura and Meseguer-Artola, 2020). This study was performed following the principles of the Declaration of Helsinki. The Colegio de Estudios Superiores de Administracion Ethics Committee ID 002 approved it on September 1, 2020.

While earlier studies have examined the connection between leadership practices and emotional intelligence in Colombian samples (Zárate Torres and Matviuk, 2012), there is currently a lack of research specifically addressing the relationship between leadership practices and happiness in Colombia. Because of Colombia's recent admission to the Organization for Economic Co-operation and Development (OECD) on April 28, 2020, Sakiru et al. (2022), the country is given more attention than samples from other nations. Since then, few empirical studies have explicitly looked at the connection between leadership and happiness, which is essential for examining how leaders behave in Colombian firms.

After being informed of the study's purpose, each participant consented. To improve data transparency and the reproducibility of results, we documented extra material following reproducible research principles (Peer et al., 2022; Peikert and Brandmaier, 2021).

We employed the Leadership Practices Inventory (LPI) and the Subjective Happiness Scale (SHS) to gather data. The four previously mentioned things make up the SHS, (1) In general, I consider myself happy; (2) Compared to most people around me, I consider myself happy (3) Some people are usually very happy. They enjoy life despite what happens and cope with most things. To what extent do you consider yourself such a person? (4) Some people are usually very unhappy. Although they are not depressed, they do not seem as happy as they would like. To what extent do you consider yourself such a person? (Lyubomirsky and Lepper, 1999). The LPI comprises a total of 30 questions. You may find more information about the LPI and its scoring guide at this URL: (https://nswactbaptists.org.au/wp-content/uploads/2023/07/Leadership-Practices-Inventory.pdf). These questions are distributed based on the following five conceptual dimensions: “challenging the process” is discussed in the first dimension (L1). The second dimension (L2) is “inspiring a shared vision”. Acquiring the ability to empower others is the third dimension (L3). The idea of “modeling the way” (L4) stands in for the fourth dimension, while “encouraging the heart” (L5) represents the fifth dimension. Next, we used the recorded responses from the participants and organized them into a standardized data set, which we then used as input for statistical analyses (Kouzes and Posner, 1990).

### 4.1 Statistical analyses

Many different kinds of analyses are used to look closely at this article. For the execution of the statistical analysis, R software, version 3.6.0 (R Core Team, 2022), was used. We used the base, tidyverse (Wickham, 2017), psych (Revelle, 2022, 2017) and lavaan (Rosseel, 2012) packages. These include traditional confirmatory factor analyses based on the ideas of Bollen (Bollen, 1989). This is important to note. A commonly used method to validate psychometric instruments is to initially conduct an exploratory factor analysis to determine if the observed structure aligns with the theoretical structure. Finally, a confirmatory factorial analysis is employed as the last empirical test. This conventional method is only suitable when the psychometric composition of the tests or questionnaires is unknown. Conducting a psychometric analysis of these exams in advance reduces the necessity of an initial exploratory factor analysis. As stated by Bollen (1989), “*in confirmatory factor analysis a model is constructed in advance, the number of latent variables is set by the analyst*” (p. 228).

To design and discover structural equation models on the happiness-leadership relationship, we used the program Ωnyx (von Oertzen et al., 2015). After Ωnyx generated the syntaxes, they were exported to RStudio for statistical testing. Additionally, we employed the R package semTable (Johnson and Kite, 2020) to make it easier to describe results using reproducible LATEX documentation (Gandrud, 2018).

## 5 Results

**Figure 1:**
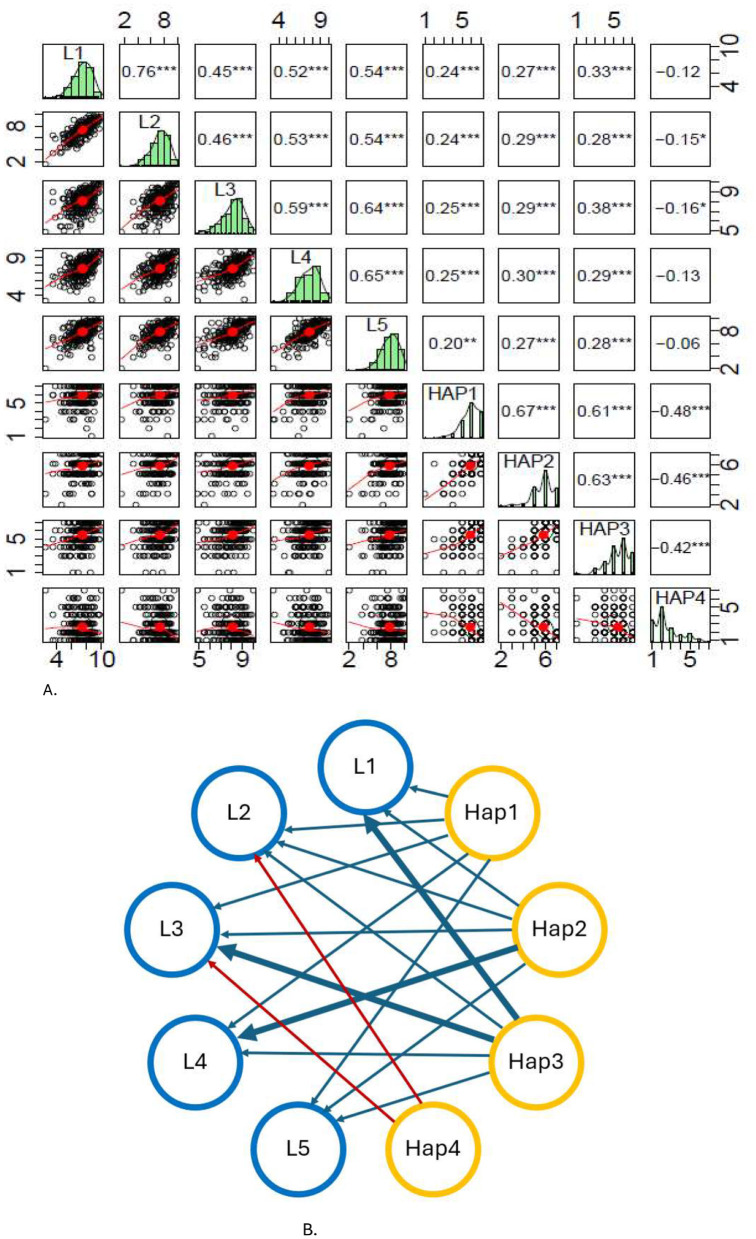
Correlations. **(A)** Spearman correlation matrix plot for the five items of leadership and the four items of happiness. **(B)** Network visualization.

There are five parts to the leadership practice inventory (L1, L2, L3, L4, and L5) and four parts to the subjective happiness scale (HAP1, HAP2, HAP3, and HAP4). To start the study, we define the univariate distribution of these parts. To examine the behavior of these discrete distributions in two dimensions, we utilized the Spearman Correlation Matrix Plot for each item, as depicted in Figure 1. As expected, all correlations among the leadership practices were statistically significant, with correlation coefficients ranging from 0.45 to 0.76 (*p*-value < 0.01). The correlations between happiness measures were statistically significant; however, their effect size was determined to be smaller. The p value is between −0.48 and 0.67, and the *p*-value is less than 0.01.

Figure 1A shows the correlations between the LPI practices and the four happiness items. The first three items of happiness show a positive correlation with the five practices of the LPI. The fourth item of happiness is “some people are usually very unhappy. Although they are not depressed, they do not seem as happy as they would like. To what extent do you consider yourself such a person?” is correlated only with two of the leadership practices, and the correlation is negative in both cases, inspiring a shared vision and enabling others to act. Having a negative correlation with this happiness item means that the less people feel unhappy, the more they want to inspire a shared vision and enable others to act.

The first three items of the SHS correlate positively with the five leadership practices of the LPI, suggesting that happiness improves leadership. On the other hand, analyzing the fourth SHS item shows that unhappiness does not affect leadership.

The relationships that confirm and reject all hypotheses are shown in Figure 1B. We now report the ensuing confirmatory factor analyses to evaluate our central hypothesis: Happiness positively influences leadership behavior.

### 5.1 Confirmatory factor analyses

It is the Subjective Happiness Scale (SHS) that is the subject of our first confirmatory factor analysis (CFA). Table 1 presents two estimates of statistical parameters. The Full Information Maximum Likelihood (FIML) approach was utilized to determine Model 1's parameters. For this strategy to work, the observed indicators must have a multivariate, continuous normal distribution. The parameters in Model 2 were estimated using Diagonally Weighted Least Squares (DWLS), which is thought to be a less restrictive estimation technique (Li, 2016). To test both models, we utilized the *lavaan* R package (Rosseel, 2012).

**Table 1:** Statistical estimated parameters for the leadership-happiness structural model.

	**Model 1**			**Model 2**		
	**Estimate (std. err.)**	**R square**	**p**	**Estimate (std. err.)**	**R square**	**p**
**Factor loadings**						
**Leadership**						
L1	0.89(0.07)^***^	0.62	0.000	0.84(0.07)^***^	0.56	0.000
L2	0.98(0.07)^***^	0.65	0.000	0.95(0.08)^***^	0.62	0.000
L3	0.65(0.06)^***^	0.50	0.000	0.68(0.05)^***^	0.55	0.000
L4	0.80(0.06)^***^	0.56	0.000	0.80(0.06)^***^	0.58	0.000
L5	0.90(0.07)^***^	0.63	0.000	0.91(0.07)^***^	0.65	0.000
**Happy**						
HAP1	0.85(0.06)^***^	0.67	0.000	0.82(0.07)^***^	0.62	0.000
HAP2	0.82(0.05)^***^	0.73	0.000	0.82(0.06)^***^	0.73	0.000
HAP3	0.89(0.07)^***^	0.52	0.000	0.95(0.07)^***^	0.60	0.000
HAP4	−0.72(0.10)^***^	0.24	0.000	−0.68(0.07)^***^	0.21	0.000
Leadership	0.54(0.09)^***^	0.23	0.000	0.55(0.06)^***^	0.23	0.000
**Fit Indices**						
χ^2^(df)	102.41(26)^***^		0.000	12.50(26)		0.988
RMSEA	0.11			0.00		
SRMR	0.04			0.04		
CFI	0.93			1.00		
TLI	0.90			1.02		

^+^Fixed parameter.

Statistical significance:

^*^p < 0.05,

^**^p < 0.01,

*** *p* < 0.001.

We present two statistical parameter estimates based on the data collected for this study in Table 1. Using the FIML estimation approach, parameters in model 1 were estimated. This method is more restrictive because it assumes that the observable indicators have a multivariate, continuous, normal distribution. The DWLS was employed in model 2 to estimate parameters.

Applying the conventional threshold values for fit indices in structural equation models (Bentler, 1990; Hu and Bentler, 1999), it is evident that the findings of model 1 in Table 1 vary among different models. Given that at least two goodness-of-fit indices of model 1 indicate that the data strongly corroborate the hypothesized theoretical structure, the conclusions drawn from the model are conclusive. However, the goodness-of-fit indexes for model 2 suggest that the data strongly support the proposed measurement model for the happiness scales.

**Figure 2:**
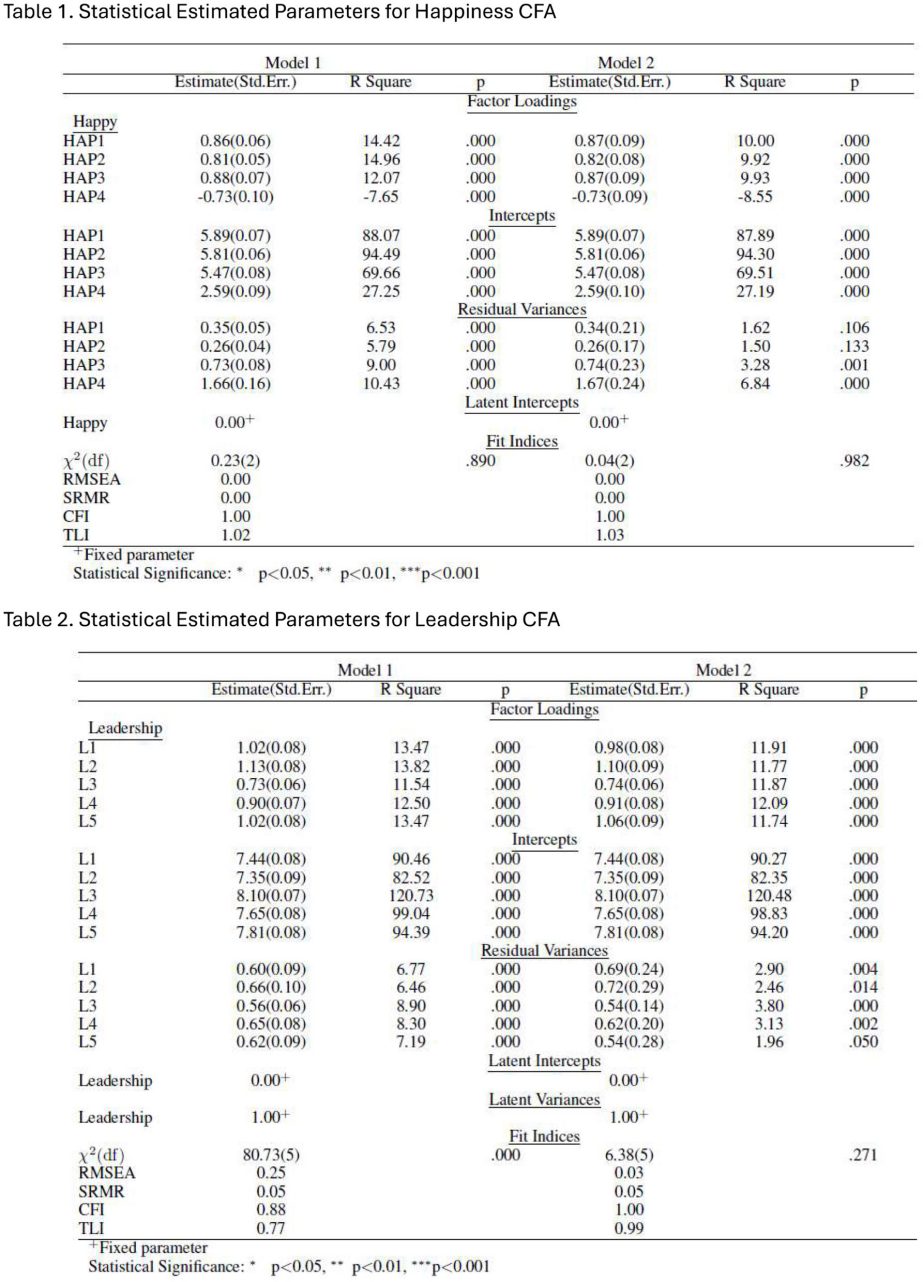
Confirmatory factor analyses.

The second set of CFA results relates to leadership. In Figure 2, we proceeded in the same way, and the results proved to be satisfactory for the second estimation model considering, as mentioned before, the traditional suggestions of cutoff criteria for fit indices for structural equation models (Bentler, 1990; Hu and Bentler, 1999).

### 5.2 Leadership-happiness structural analyses

**Figure 3:**
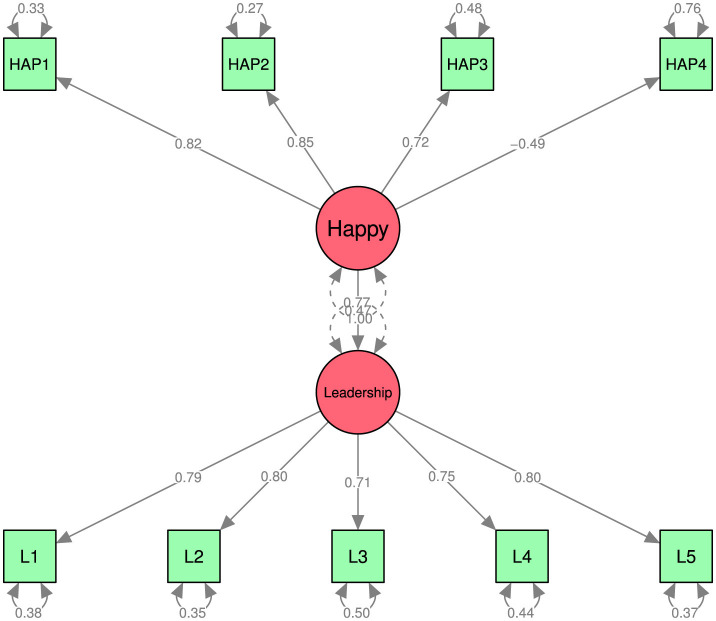
Structural leadership-happiness model.

Once the psychometric structure of our two conceptions has been established, we will turn our focus to how they relate structurally (as shown in Figure 3 and described in Table 1). All factor loadings remained statistically significant using both parametric estimating procedures, as seen in Figure 2. However, the connection between happiness and leadership was found to be moderately weak, regardless of whether the FIML estimation method (Est = 0.54, standard error = 0.09, **p**-value < 0.00, R^2^ = 0.23) or the DWLS estimation method (Est = 0.55, standard error = 0.06, *p*-value < 0.00, R^2^ = 0.23) was used.

The FIML estimation method was employed to calculate the parameters of model 1. This method is deemed more constrained because it implies that the observable indicators conform to a multivariate, continuous normal distribution. The DWLS was used to estimate the parameters in model 2 (Li, 2016) as a less constrained estimation technique.

At least two goodness-of-fit indexes show that the results from model 1 are inconclusive, which suggests that the data does not support the proposed theoretical framework. Conversely, all goodness-of-fit indexes derived from model 2 indicate that the data supports the proposed theoretical model (Bentler, 1990; Hu and Bentler, 1999) and indicate that the model fits the data. However, the FIML estimation method (Est = 0.54, standard error = 0.09, *p*-value < 0.00) or the DWLS estimation method (Est = 0.55, standard error = 0.06, **p**-value < 0.00) both revealed that the happiness-leadership relationship is moderately weak because in the FIML and the DWLS estimation methods is twenty percent of the explained variance (R^2^ = 0.23).

The results show a good fit of the empirical data concerning the theoretical model (Bentler, 1990; Hu and Bentler, 1999). Between model 1 (FIML) and model 2 (DWLS), the variation of the results is minimal and occurs in the RMSEA (root mean squared error of approximation), which goes from 0.11 in model 1 to 0.00 in model 2, a model that adequately fits the sample (Lai and Green, 2016). All fit indices are more than adequate to indicate that the model fits the data. The results indicate that all the hypotheses are confirmed.

## 6 Discussion and implications

The study shows that all the variables evaluated about Happiness (Hap1, Hap2, and Hap3) have a direct and positive influence on the five leadership practices of the LPI. Therefore, it is essential for human talent offices to formulate happiness indicators that contribute to developing leadership programs in the institutions.

There is an association between leadership and happiness at work in organizational learning capability and organizational facilitators (Setiawan et al., 2020), consistent with what was found in this research between leadership practices and happiness variables. The findings were confirmed with the quantum leadership model (Ahmadiyan et al., 2022), which found a statistically significant correlation between this type of leadership, organizational health, and happiness. Therefore, institutions should include measures of self-perception of happiness in the evaluation processes of leaders to detect early needs for intervention, mentoring, and accompaniment.

Similarly, this research shows that inspiring a shared vision and encouraging the heart correlates positively with the elements of happiness assessed. Similar results suggest that employees can be happier when the leader is a good listener (Isa et al., 2019) and that servant leadership has a positive relationship with organizational enablers and work happiness (Vallina and Guerrero, 2018). These results reinforce the findings of this research as related to the practices of enabling others to act and challenging the process understood as organizational enablers. In this sense, leaders must be aware of their perception of happiness's impact on their teams and the potential influence on the development of the organizational climate.

Consistent with H_2_ of this study, the positive relationships between perceived happiness and the five leadership practices (Srivastava et al., 2022) found a relationship between happiness and prosocial behavior (cooperative acts based on people's principles) are consistent with the findings of this study, where the relationship with greater intensity is found between the perception of happiness and leadership practice 4, in which the leader models by example the transparency of his principles and the consistency of his behavior. Therefore, leaders must understand that their happiness strengthens the skills that produce an environment of team trust and increases motivation, potentially developing happier teams. Further studies - complementary to happiness–can strengthen the conceptual frameworks of leadership.

Many studies correlate leadership and its different typologies with employee happiness and their perception of wellbeing (Salas-Vallina, 2018; Carrillo et al., 2023; Romão et al., 2022; Sari and Prasadjaningsih, 2023). However, understanding happiness as an independent variable and its influence on leadership practices is an original contribution, extending previous research on these topics, which contributes to the theoretical foundation of the topic, providing a complementary and bidirectional approach to the relationship between leadership and wellbeing. On the other hand, this study provides theoretical elements of variables, such as happiness, that positively influence transformational leadership, which opens the door to further research that seeks to identify other variables that contribute to the development of this type of leadership.

We recommend incorporating other variables into the study for subsequent research. For instance, supervisor experience–managing and guiding others–may be instructive since it offers an alternative perspective on the relationship between happiness and leadership. We are well aware that people who have worked as supervisors for a more extended period may be able to control their conduct and happiness when they need to argue with coworkers and/or convince peers, superiors, and collaborators.

## 7 Conclusions

The study shows that happiness positively influences transformational leadership traits and behaviors. Specifically, three aspects of happiness positively correlate with the five practices of the Leadership Practices Inventory (LPI), suggesting that happiness improves leadership. Confirmatory factor analyses strongly support the proposed measurement model for the happiness scales. Furthermore, structural analyses show a good fit of the empirical data concerning the theoretical model.

The research offers valuable implications for Latin American companies, as it provides data that demonstrates how leader happiness strengthens key transformational leadership practices–such as inspiring, empowering, and modeling by example–which, in turn, promote motivation, commitment, and organizational performance. The findings encourage leaders of organizations in the Global South to incorporate emotional wellness strategies, such as promoting a sense of purpose and individual recognition, to enhance their employees' wellbeing. The adoption of these approaches not only promotes healthier and more sustainable work environments but also aligns with ethical and humanistic leadership practices rooted in the region (Robertson et al., 2011; López Pérez et al., 2023)

Regarding future theoretical developments, this study highlights the need to investigate mediating variables that explain the relationship between happiness and leadership. Variables such as emotional intelligence (García-Salirrosas et al., 2025), labor inclusion (Jha et al., 2024), and psychological safety (Edmondson, 1996; Newman et al., 2017) could be explored through longitudinal or quasi-experimental studies over time.

Similarly, it would be enriching to delve deeper into specific contexts, such as family businesses in the Latin American context, to compare how these mechanisms are activated in organizations with different structures and cultures (Ramirez-Lozano et al., 2023). In practice, organizations in the Global South can utilize this study to implement leadership development programs that incorporate training in subjective wellbeing, meditation, active pauses, and regular emotional recognition. Likewise, the application of tools validated in Spanish–such as the Subjective Happiness Scale–facilitates the diagnosis of leaders' wellbeing and the evaluation of specific interventions (Extremera and Fernández-Berrocal, 2014). The integration of these techniques can improve job satisfaction indicators, reduce turnover, and enhance internal cohesion, as evidenced by experiences in Peru and Chile related to organizational happiness (Silva Munar et al., 2020; García-Salirrosas et al., 2025).

This article can contribute to the redesign and/or adjustment of public and business policies that promote integral wellbeing, such as healthy work environments, by recognizing that the leader's happiness is an antecedent and not only a consequence of his or her leadership; it leads to focus on the measurement of the emotional capital of leadership (Luthans, 2002; Avey et al., 2010). Along these lines, companies in the region can integrate happiness indicators in balanced scorecards, accompanied by practices that promote organizational justice (Jha et al., 2024), labor inclusion, work-life balance (Medina-Garrido et al., 2024), generating positive impacts on both wellbeing and sustainable performance.

This study highlights the importance of happiness in today's organizations. The results obtained indicate that individuals who demonstrate greater happiness exhibit better leadership behaviors, which, in turn, favors productivity. In other words, leaders who experience and promote happiness tend to be more effective in their role, which translates into better performance from their teams and, ultimately, the organization.

The research underscores the relevance of considering leaders' emotional wellbeing as a key factor for organizational success. Organizations can cultivate more effective leadership and improve their overall productivity by fostering a positive work environment where happiness is valued. This approach suggests that happiness is an aspect of individual wellbeing and essential to effective leadership and organizational performance.

## Data Availability

The datasets presented in this study can be found in online repositories. The names of the repository/repositories and accession number(s) can be found below: https://github.com/rodzarate/Leadership-Happiness.

## References

[B1] Abdullah A. G. K. Ling Y. Ping C. S. (2017). Workplace happiness, transformational leadership and affective commitment. Adv. Sci. Lett. 23, 2872–2875. 10.1166/asl.2017.7588

[B2] Ackaradejruangsri P. Mumi A. Rattanapituk S. Pakhunwanich P. (2023). Exploring the determinants of young inclusive leadership in thailand: research taxonomy and theoretical framework. J. Knowl. Econ. 14, 3696–3723. 10.1007/s13132-022-01017-7 40479243 PMC8964255

[B3] Ahmadiyan Z. Zareiyan A. Azizi M. Jahandari P. Zargar Balaye Jame S. Ganjizadeh H. (2022). Mediating role of happiness in the relationship between quantum leadership and organizational health at AJA university of medical sciences. J. Mil. Med. 23, 802–811. Available online at: https://militarymedj.bmsu.ac.ir/article_1001113.html?lang=en

[B4] Alarcón R. (2006). Desarrollo de una escala factorial para medir la felicidad. J Psychol. 40, 99–106.

[B5] Alarcón R. (2015). La idea de la felicidad. Apuntes de Ciencia & Sociedad 5:2. 10.18259/acs.2015002

[B6] Alquwez N. Cruz J. P. Alotaibi N. S. Alshammari F. (2021). Validity and reliability of the subjective happiness scale arabic version among saudi working women. J. Taibah Univers. Med. Sci. 16:835–842. 10.1016/j.jtumed.2021.05.010 34899127 PMC8626824

[B7] Avey J. Luthans F. Smith R. M. Palmer N. F. (2010). Impact of positive psychological capital on employee well being over time. J. Occup. Health Psychol. 15:17–28. 10.1037/a0016998 20063956

[B8] Bass B. Riggio R. (2006). Transformational Leadership. Oxfordshire: Taylor & Francis.

[B9] Bentler P. (1990). Comparative fit indexes in structural models. Psychol. Bull. 107, 238–246. 10.1037//0033-2909.107.2.238 2320703

[B10] Blomme R. J. (2012). Leadership, complex adaptive systems, and equivocality: The role of managers in emergent change. Organizat. Managem. J. 9, 4–19. 10.1080/15416518.2012.666946

[B11] Blomme R. J. Kodden B. Beasley-Suffolk A. (2015). Leadership theories and the concept of work engagement: creating a conceptual framework for management implications and research. J. Manag. Organ. 21, 125–144. 10.1017/jmo.2014.71

[B12] Bollen K. A. (1989). Structural Equations with Latent Variables. New York: Wiley and Sons.

[B13] Burns J. (1978). Leadership. New York: Harper and Row.

[B14] Canal A. I. Ovalles-Toledo L. V. Sandoval L. A. Valdez Palazuelos O. (2023). Liderazgo transformacional y su relación con la felicidad en el trabajo: Empresas sinaloenses del sector agroindustrial. Revista De Ciencias Sociales 29, 79–94.

[B15] Carrillo A. I. C. Ovalles L. Barraza L. A. S. Palazuelos O. V. (2023). Liderazgo transformacional y su relación con la felicidad en el trabajo: Empresas sinaloenses del sector. Revista de ciencias sociales 29, 79–94. Available online at: https://dialnet.unirioja.es/servlet/articulo?codigo=8822428

[B16] Cassar L. Meier S. (2018). Nonmonetary incentives and the implications of work as a source of meaning. J. Econ. Perspect., 32, 215–238. 10.1257/jep.32.3.215

[B17] Charles-Leija H. Castro C. G. Toledo M. Ballesteros-Valdés R. (2023). Meaningful work, happiness at work, and turnover intentions. Int. J. Environ. Res. Public Health 20, 3565. 10.3390/ijerph20043565 36834260 PMC9963286

[B18] Chien C.-L. Chen P.-L. Chu P.-J. Wu H.-Y. Chen Y.-C. Hsu S.-C. (2020). The chinese version of the subjective happiness scale: validation and convergence with multidimensional measures. J. Psychoeduc. Assess. 38, 222–235. 10.1177/0734282919837403

[B19] Csikszentmihalyi M. (1990). Flow: The Psychology of Optimal Experience. New York: HarperPerennial.

[B20] Díaz E. R. López K. M. D. Watanabe E. D. (2020). Adaptación del inventario de prácticas de liderazgo con estudiantes mexicanos de posgrado. Revista del Centro de Investigación de la Universidad la Salle 14, 95–118.

[B21] DiMaria C. H. Peroni C. Sarracino F. (2020). Happiness matters: Productivity gains from subjective well-being. J. Happiness Stud. 21, 139–160. 10.1007/s10902-019-00074-1

[B22] Durán M. Castañeda D. (2015). Relación entre liderazgo transformacional y transaccional con la conducta de compartir conocimiento en dos empresas de servicios. Acta Colombiana de Psicología 18, 135–147. 10.14718/ACP.2015.18.1.13

[B23] Edmondson A. C. (1996). Learning from mistakes is easier said than done: group and organizational influences on the detection and correction of human error. J. Appl. Behav. Sci., 32, 5–28. 10.1177/0021886396321001

[B24] Emmanuel S. Valley C. A. (2022). A qualitative case study of exemplary principal leadership in the united states virgin islands: an application of kouzes and posner's five practices of exemplary leadership. J. Res. Leadersh. Educ. 17, 243–264. 10.1177/1942775121990054

[B25] Extremera N. Fernández-Berrocal P. (2014). The subjective happiness scale: translation and preliminary psychometric evaluation of a spanish version. Soc. Indic. Res. 119, 473–481. 10.1007/s11205-013-0497-2

[B26] Feliu-Soler A. de Diego-Adeli no J. Luciano J. V. Iraurgi I. Alemany C. Puigdemont D.. (2021). Unhappy while depressed: Examining the dimensionality, reliability and validity of the subjective happiness scale in a spanish sample of patients with depressive disorders. Int. J. Environ. Res. Public Health 18:10964. 10.3390/ijerph182010964 34682709 PMC8535987

[B27] Fisher C. D. (2010). Happiness at work. Int. J. Manag. Rev. 12:384–412. 10.1111/j.1468-2370.2009.00270.x

[B28] Francia V. H. R. Gurieva N. Velázquez S. C. A. (2012). Liderazgo sin moralidad: Un estudio correlacional entre el inventario de prácticas de liderazgo (IPL) y el juicio moral. Investigación Administrativa 110, 7–17.

[B29] Gandrud C. (2018). Reproducible Research with R and RStudio. Boca Raton, FL: Chapman and Hall/CRC.

[B30] García M. (2016). Bienestar Emocional en Educación: Empecemos por los Maestros (PhD thesis). Universidad de Murcia, Murcia, Spain.

[B31] García-Salirrosas E. E. Yong-Chung F. E. Jauregui-Arroyo R. R. Escobar-Farfán M. Acevedo-Duque Á. (2025). Impact of leader behavior on employee experience and job satisfaction in educational institutions. Administ. Sci.15:119. 10.3390/admsci15040119

[B32] Hancock A. J. Gellatly I. R. Walsh M. M. Arnold K. A. Connelly C. E. (2021). Good, bad, and ugly leadership patterns: Implications for followers' work-related and context-free outcomes. J. Manage. 49, 640–676. 10.1177/01492063211050391 36484084 PMC9720459

[B33] Hardwicke T. Thibault R. Kosie J. Wallach J. Kidwell M. Ioannidis J. (2022). Estimating the prevalence of transparency and reproducibility-related research practices in psychology (2014–2017). Perspect. Psychol. Sci. 17, 239–251. 10.1177/1745691620979806 33682488 PMC8785283

[B34] Harris K. J. Kacmar K. M. (2006). Too much of a good thing: The curvilinear effect of leader-member exchange on stress. J. Soc. Psychol. 146, 65–84. 10.3200/SOCP.146.1.65-84 16480122

[B35] Helland M. R. Winston B. E. (2005). Towards a deeper understanding of hope and leadership. J. Leaders. Organizat. Stud. 12, 42–54. 10.1177/107179190501200204

[B36] Helliwell J. Layard R. Sachs J. (2017). World Happiness Report 2017. New York, NY: Sustainable Development Solutions Network.

[B37] Helliwell J. Layard R. Sachs J. (2018). World Happiness Report 2018. New York, NY: Sustainable Development Solutions Network.

[B38] Helliwell J. F. Layard R. Sachs J. De Neve J. E. (2020). World Happiness Report 2020. New York, NY: Sustainable Development Solutions Network.

[B39] Hu L. Bentler P. M. (1999). Cutoff criteria for fit indexes in covariance structure analysis: Conventional criteria versus new alternatives. Struct. Equ. Model. 6, 1–55. 10.1080/1070551990954011836787513

[B40] Institute of Leadership & Management (2011). The Pursuit of Happiness: Positivity and Performance Among UK Managers. London: Institute of Leadership & Management.

[B41] Isa K. Tenah S. S. Atim A. Jam N. A. M. (2019). Leading happiness: Leadership and happiness at a workplace. Int. J. Recent Technol. Eng. 8, 6551–6553. 10.35940/ijrte.C5299.09831938902408

[B42] Jha I. N. Pal D. Sarkar S. (2024). Unlocking the secret to happiness at work: the power of inclusive leadership, organizational justice and workplace inclusion. J. Manag. Dev. 43, 200–221. 10.1108/JMD-04-2023-0136

[B43] Johnson P. Kite B. (2020). “semTable: structural equation modeling tables,” in R Package Version 1.8.

[B44] Joseph S. Linley P. A. Harwood J. Lewis C. A. McCollam P. (2004). Rapid assessment of well-being: the short Depression-Happiness scale (SDHS). Psychol. Psychother. 77, 463–478. 10.1348/1476083042555406 15588455

[B45] Junça-Silva A. Camaz A. (2023). A longitudinal approach to disentangle how conscientiousness creates happy people: The mediating role of self-leadership and the moderating role of perceived leadership effectiveness. Heliyon 9:e16893. 10.1016/j.heliyon.2023.e16893 37360082 PMC10285127

[B46] Karakasidou E. Pezirkianidis C. Stalikas A. Galanakis M. (2016). Standardization of the subjective happiness scale (SHS) in a greek sample. Psychology 7, 1753–1765. 10.4236/psych.2016.714164

[B47] Kılıç M. Günsel A. (2019). The dark side of the leadership: the effects of toxic leaders on employees. Eur. J. Soc. Sci. 2, 51–56. 10.26417/ejss-2019.v2i2-64

[B48] Kouzes J. M. Posner B. Z. (1987). The Leadership Challenge. New York: John Wiley & Sons.

[B49] Kouzes J. M. Posner B. Z. (1990). Leadership Practices: An Alternative to the Psychological Perspective. Greensboro, NC: Leadership Press.

[B50] Kouzes T. K. Posner B. Z. (2019). Influence of managers' mindset on leadership behavior. Leaders. Organiz. Dev. J. 40, 829–844. 10.1108/LODJ-03-2019-0142

[B51] Lai K. Green S. B. (2016). The problem with having two watches: Assessment of fit when rmsea and cfi disagree. Multivariate Behav. Res. 51, 220–239. 10.1080/00273171.2015.1134306 27014948

[B52] Levin K. A. (2006). Study design IV: Cross-sectional studies. Evid. Based Dent. 7, 24–25. 10.1038/sj.ebd.6400375 16557257

[B53] Lewis C. A. Francis L. J. Zieberts H. G. (2002). The internal consistency reliability and construct validity of the german translation of the oxford happiness inventory. N. Am. J. Psychol. 4, 211–220. Available online at: www.researchgate.net/publication/313048485_The_internal_consistency_reliability_ and_construct_validity_of_the_German_translation_of_the_Oxford_Happiness">http://www.researchgate.net">www.researchgate.net/publication/313048485_The_internal_consistency_reliability_ and_construct_validity_of_the_German_translation_of_the_Oxford_Happiness

[B54] Li C.-H. (2016). The performance of ml, dwls, and uls estimation with robust corrections in structural equation models with ordinal variables. Psychol. Methods 21:369. 10.1037/met0000093 27571021

[B55] López Pérez C. P. Vieira Salazar J. A. Echeverri Rubio A. (2023). El liderazgo y su influencia en la felicidad en el trabajo: una revisión narrativa de la literatura. Cuad. Adm., 39:e4112627. 10.25100/cdea.v39i75.12627

[B56] Lorente L. Vera M. (2010). Las organizaciones saludables: “el engagement en el trabajo”. gestión práctica de riesgos laborales: Integración y desarrollo de la gestión de la prevención. 73:16–20.

[B57] Luthans F. (2002). Positive organizational behavior: Developing and managing psychological strengths. Acad. Managem. Execut. 16, 57–72. 10.5465/ame.2002.6640181

[B58] Lyubomirsky S. King L. Diener E. (2005a). The benefits of frequent positive affect: does happiness lead to success? Psychol. Bull. 131, 803–855. 10.1037/0033-2909.131.6.803 16351326

[B59] Lyubomirsky S. Lepper H. S. (1999). A measure of subjective happiness: Preliminary reliability and construct validation. Soc. Indic. Res. 46, 137–155. 10.1023/A:100682410004128181474

[B60] Lyubomirsky S. Sheldon K. M. Schkade D. (2005b). Pursuing happiness: The architecture of sustainable change. Rev. Gen. Psychol. 9, 111–131. 10.1037/1089-2680.9.2.111

[B61] Lyubomirsky S. Tucker K. L. (1998). Implications of individual differences in subjective happiness for perceiving, interpreting, and thinking about life events. Motiv. Emot. 22, 155–186. 10.1023/A:1021396422190

[B62] Martínez-Conesa I. Iglesias-Sánchez P. Jambrino-Maldonado C. Fernández-Díaz E. (2024). “Do happy leaders have a different leadership style?,” in 8 International Academic and Professional Congress on Happiness (Málaga: 8 International Academic and Professional Congress on Happiness).

[B63] Medina-Garrido J. A. Biedma-Ferrer J. M. Ramos-Rodriguez A. R. (2024). Relationship between work-family balance, employee well-being and job performance. arXiv. 10.48550/arXiv.2401.13683

[B64] Mendoza-Ocasal D. Castillo-Jiménez R. Navarro E. Ramírez J. (2021). Measuring workplace happiness as a key factor for the strategic management of organizations. Pol. J. Manag. Stud. 24, 292–306. 10.17512/pjms.2021.24.2.18

[B65] Newman A. Donohue R. Eva N. (2017). Psychological safety: A systematic review of the literature. Hum. Resour. Manag. Rev. 27, 521–535. 10.1016/j.hrmr.2017.01.001

[B66] Northouse P. G. (2016). Leadership: Theory and Practice. Thousand Oaks, CA: SAGE Publications.

[B67] Nú nez-Barriopedro E. Ravina-Ripoll R. Ahumada-Tello E. (2020). Happiness perception in Spain, a SEM approach to evidence from the sociological research center. Qual. Quant. 54, 761–779. 10.1007/s11135-019-00955-w

[B68] Olvera-Calderón J. LLorens-Gumbau S. Acosta-Antognoni H. Salanova-Soria M.. (2017). El liderazgo transformacional y la confianza como antecedentes del desempe no en equipo en el ámbito sanitario. Ann. Psychol. 33, 365–375. 10.6018/analesps.33.2.237291

[B69] Ortiz F. R. Paiva S. M. Pordeus I. A. Ardenghi T. M. (2021). Psychometric properties and longitudinal measurement invariance of the Brazilian version of the subjective happiness scale in adolescents. J. Clini. Transl. Res. 7:234. Available online at: https://pmc.ncbi.nlm.nih.gov/articles/PMC8177854/34104826 PMC8177854

[B70] Pangarso A. Pradana M. Widodo A. Putera K. (2019). Bank's employees happiness factor analysis (a study in bank btn harmoni branch, jakarta, indonesia). J. Adv. Res. Dynam. Cont. Syst. 11, 750–758. 10.5373/JARDCS/V11SP12/20193267

[B71] Pavot W. Diener E. (2009). “Review of the satisfaction with life scale,” in Assessing Well-Being (Dordrecht: Springer Netherlands), 101–117.

[B72] Peer L. Biniossek C. Betz D. Christian T.-M. (2022). “Reproducible research publication workflow: a canonical workflow framework and fair digital object approach to quality research output,” in Data Intelligences, 1–14.

[B73] Peikert A. Brandmaier A. M. (2021). A reproducible data analysis workflow with R Markdown, Git, Make, and Docker. Quant. Comput. Methods Behav. Sci. 1:3763. 10.5964/qcmb.3763

[B74] Platón Lan, C. E. del Pozo Ortiz A. Gual C. (2016). Dialogos IV.: Republica, volume 25. Barcelona: RBA Libros.

[B75] Posner B. Z. Kouzes J. M. (1988). Development and validation of the leadership practices inventory. Educ. Psychol. Meas. 48, 483–496. 10.1177/0013164488482024

[B76] Powell M. Bateman C. J. Gerasimova D. Bateman A. Peltzer K. (2020). Psychometric properties of the subjective happiness scale in four asian countries. J. Well-Being Assessm. 4, 495–509. 10.1007/s41543-021-00045-5

[B77] Quezada L. Landero R. González M. T. (2016). A validity and reliability study of the subjective happiness scale in mexico. J. Happin. Well-Being 4, 90–100. Available online at: https://jhwbjournal.com/uploads/files/648458ef95b75d3e12f241a2c6f98015.pdf23607679

[B78] R Core Team (2022). R: A Language and Environment for Statistical Computing. Vienna: R Foundation for Statistical Computing.

[B79] Ramirez-Lozano J. Pe naflor-Guerra R. Sanagustín-Fons V. (2023). Leadership, communication, and job satisfaction for employee engagement and sustainability of family businesses in latin america. Adm. Sci. 13:137. 10.3390/admsci13060137

[B80] Ravina Ripoll R. Marchena Domínguez J. Monta nés Del Rio M. Á. (2019). Happiness management en la época de la industria 4.0. Retos 9:189–202. 10.17163/ret.n18.2019.01

[B81] Ravina Ripoll R. Romero-Rodríguez L. M. Ahumada-Tello E. (2022). Guest editorial: Happiness management: key factors for sustainability and organizational communication in the age of industry 4.0. Corp. Gov. 22, 449–457. 10.1108/CG-05-2022-576

[B82] Ravina-Ripoll R. Romero-Rodríguez L. M. Ahumada-Tello E. (2022). Workplace happiness as a trinomial of organizational climate, academic satisfaction and organizational engagement. Corp. Gov. 22, 474–490. 10.1108/CG-12-2020-0532

[B83] Revelle W. (2022). “psych: Procedures for Psychological, Psychometric, and Personality Research. Northwestern University, Evanston, Illinois,” in R Package Version 2.2.5.

[B84] Revelle W. R. (2017). psych: Procedures for Personality and Psychological Research [Software]. Evanston, IL: Northwestern University.

[B85] Rienzi L. (2025). Entregando felicidad: el liderazgo como motor de éxito. California, USA. ADEN International Business School.

[B86] Rivera D. A. Carrillo S. M. Forgiony F. Nuván I. L. Rozo A. C. (2018). Cultura organizacional, retos y desafíos para las organizaciones saludables. Revista Espacios 38, 1–14. Available online at: https://www.revistaespacios.com/a18v39n22/a18v39n22p27.pdf

[B87] Robertson I. T. Cooper C. L. Johnson S. (2011). Well-being: Productivity and Happiness at Work, volume 3. Basingstoke: Palgrave Macmillan.

[B88] Robles Francia V. H. Contreras Torres F. Barbosa Ramírez D. Juárez Acosta F. (2013). Liderazgo en directivos colombianos vs. mexicanos. un estudio comparativo. Investigación y desarrollo 21, 395–418. Available online at: https://www.redalyc.org/pdf/268/26828939004.pdf

[B89] Rodríguez-Ardura I. Meseguer-Artola A. (2020). A pls-neural network analysis of motivational orientations leading to facebook engagement and the moderating roles of flow and age. Front. Psychol. 11:1869. 10.3389/fpsyg.2020.01869 32903790 PMC7438855

[B90] Rom ao S. Ribeiro N. Gomes D. R. Singh S. (2022). The impact of leaders' coaching skills on employees' happiness and turnover intention. Admin. Sci. 12:84. 10.3390/admsci12030084

[B91] Rosseel Y. (2012). lavaan: an R package for structural equation modeling. J. Stat. Softw. 48, 1–36. 10.18637/jss.v048.i02

[B92] Rowan A. N. (2023). “World happiness report 2023,” in WellBeing News, Vol. 5. Available online at: https://www.wellbeingintlstudiesrepository.org/wbn/vol5/iss3/1

[B93] Russell B. (1936). La conquista de la felicidad (Juan M. Ibeas, Trad.). Santiago de Chile: Ediciones Ercilla. (Traducción original copyright 2000).

[B94] Sakiru S. Gil-Alana L. Gonzalez-Blanch M. (2022). Persistence of economic complexity in OECD countries. Physica A: Statist. Mech. Appl. 603:127860. 10.1016/j.physa.2022.127860

[B95] Salanova M. Llorens S. (2009). Exposición a la tecnología de la información y la comunicación y su relación con el engagement. Ciencia y Trabajo 11, 55–62.

[B96] Salas-Vallina A. (2018). Liderazgo femenino y felicidad en el trabajo: el papel mediador del intercambio líder-colaborador. Búsqueda 5, 146–164. 10.21892/01239813.417

[B97] Salas-Vallina A. López-Cabrales Á. Alegre J. Fernández R. (2017). On the road to happiness at work (HAW). Pers. Rev. 46, 314–338. 10.1108/PR-06-2015-0186

[B98] Salas-Vallina A. Simone C. Fernández-Guerrero R. (2020). The human side of leadership: Inspirational leadership effects on follower characteristics and happiness at work (HAW). J. Bus. Res. 107, 162–171. 10.1016/j.jbusres.2018.10.044

[B99] Sánchez I. K. Ríos M. J. Cajas V. E. Tanqueño O. P. (2021). Liderazgo positivo en organizaciones saludables. Revista Venezolana de Gerencia 26, 544–563. 10.52080/rvgluz.27.95.738744586

[B100] Sari D. A. A. Prasadjaningsih M. O. (2023). Millenial employees perception related to workload, work life balance, challenge leadership on happiness at work case study kuningan area south jakarta. J. Account. Finance Managem. 4, 210–219. 10.38035/jafm.v4i2.219

[B101] Seligman M. E. P. Steen T. A. Park N. Peterson C. (2005). Positive psychology progress: empirical validation of interventions. Am. Psychol. 60, 410–421. 10.1037/0003-066X.60.5.410 16045394

[B102] Setiawan R. Eliyana A. Suryani T. Handojo A. (2020). Maximizing happiness at work: the best practices of transformational leadership at food and beverage start-up business in Indonesia. Sys. Rev. Pharm. 11, 1265–1271. 10.31838/srp.2020.12.186

[B103] Silva Munar J. L. De Juana-Espinosa S. Martínez-Buelvas L. Vecchiola Abarca Y. Orellana Tirado J. (2020). Organizational happiness dimensions as a contribution to sustainable development goals: a prospective study in higher education institutions in chile, colombia and spain. Sustainability 12:10502. 10.3390/su122410502

[B104] Sousa L. M. M. Marques-Vieira C. M. A. Severino S. S. P. Pozo-Rosado J. L. José H. M. G. (2017). Validación de la “subjective happiness scale” en personas con enfermedad renal crónica. Enfermería Global 16, 38–70. 10.6018/eglobal.16.3.266571

[B105] Srivastava S. Mendiratta A. Pankaj P. Misra R. Mendiratta R. (2022). Happiness at work through spiritual leadership: a self-determination perspective. Empl. Relat. 44, 972–992. 10.1108/ER-08-2021-0342

[B106] Steel P. Schmidt J. Shultz J. (2008). Refining the relationship between personality and subjective well-being. Psychol. Bull. 134, 138–161. 10.1037/0033-2909.134.1.138 18193998

[B107] Tkach C. Lyubomirsky S. (2006). How do people pursue happiness?: Relating personality, happiness-increasing strategies, and well-being. J. Happiness Stud. 7, 183–225. 10.1007/s10902-005-4754-1

[B108] Urzúa A. Cortés E. Vega S. Prieto L. Tapia K. (2009). Propiedades psicométricas del cuestionario de auto reporte de la calidad de vida KIDSCREEN-27 en adolescentes chilenos. Terapia Psicológica 27, 83–92. 10.4067/S0718-4808200900010000825694287

[B109] Vallina A. S. Guerrero R. F. (2018). The human side of leadership: Exploring the relationship between servant leadership, organisational facilitators and happiness at work. Int. J. Environm. Health 9, 131–150. 10.1504/IJENVH.2018.09277235009967

[B110] Van Maurik J. (2001). Writers on Leadership. London: Penguin UK.

[B111] Visser V. van Knippenberg D. van Kleef G. Wisse B. (2013). How leader displays of happiness and sadness influence follower performance: Emotional contagion and creative versus analytical performance. Leadersh. Q. 24, 172–188. 10.1016/j.leaqua.2012.09.003

[B112] von Oertzen T. Brandmaier A. M. Tsang S. (2015). Structural equation modeling with Ωnyx. Struct. Equ. Model. 22, 148–161. 10.1080/10705511.2014.93584233892413

[B113] Wickham H. (2017). tidyverse: Easily Install and Load the Tidyverse (Version 1.2.1) [R package]. Retrieved from: https://CRAN.R-project.org/package=tidyverse

[B114] Wolor C. W. Ardiansyah A. Rofaida R. Nurkhin A. Rababah M. (2022). Impact of toxic leadership on employee performance. Health Psychol. Res. 10:57551. 10.52965/001c.57551 36540087 PMC9760724

[B115] Zager Kocjan G. Jose P. E. Sočan G. Avsec A. (2022). Measurement invariance of the subjective happiness scale across countries, gender, age, and time. Assessment 29, 826–841. 10.1177/1073191121993558 33576241 PMC9047108

[B116] Zanon C. Fabretti R. R. Martins J. Z. Heath P. J. (2022). Adaptation of the steen happiness index (SHI) to brazil: a comparison of the psychometric properties of the shi and the subjective happiness scale. Assessment 29, 1597–1610. 10.1177/10731911211024354 34142565

[B117] Zárate Torres R. A. Matviuk S. (2012). Inteligencia emocional y prácticas de liderazgo en las organizaciones colombianas. Cuadernos de Administración 28, 91–104. 10.25100/cdea.v28i47.76

[B118] Zárate-Torres R. Correa J. C. (2023). How good is the Myers-Briggs type indicator for predicting leadership-related behaviors? Front. Psychol. 14:940961. 10.3389/fpsyg.2023.940961 36936015 PMC10017728

